# Postoperative Osteoporosis in Subjects with Morbid Obesity Undergoing Bariatric Surgery with Gastric Bypass or Sleeve Gastrectomy

**DOI:** 10.3390/nu15061302

**Published:** 2023-03-07

**Authors:** Jan O. Aaseth, Jan Alexander

**Affiliations:** 1 Department of Research, Innlandet Hospital Trust, P.O. Box 104, N-2381 Brumunddal, Norway; 2 Faculty of Health and Social Sciences, Inland Norway University of Applied Sciences, N-2418 Elverum, Norway; 3 Norwegian Institute of Public Health, P.O. Box 222, N-0213 Oslo, Norway

**Keywords:** obesity, bariatric surgery, osteoporosis, nutritional supplements, micronutrients, trace elements, vitamins, gastric bypass, gastric sleeve

## Abstract

Obesity has become a worldwide epidemic accompanied by adverse health effects. The limited efficiency of traditional weight reduction regimens has led to a substantial increase in the use of bariatric surgery. Today, sleeve gastrectomy (SG) and Roux-en-Y-gastric bypass (RYGB) are the most used procedures. The present narrative review focuses on the risk of developing postoperative osteoporosis and summarizes some of the most relevant micronutrient deficiencies associated with RYGB and SG. Preoperatively, the dietary habits of obese individuals might lead to precipitated deficiencies in vitamin D and other nutrients affecting bone mineral metabolism. Bariatric surgery with SG or RYGB can aggravate these deficiencies. The various surgical procedures appear to affect nutrient absorption differently. Being purely restrictive, SG may particularly affect the absorption of vitamin B_12_ and also vitamin D. In contrast, RYGB has a more profound impact on the absorption of fat-soluble vitamins and other nutrients, although both surgical methods induce only a mild protein deficiency. Despite adequate supplementation of calcium and vitamin D, osteoporosis may still occur after the surgery. This might be due to deficiencies in other micronutrients, e.g., vitamin K and zinc. Regular follow-ups with individual assessments and nutritional advice are indispensable to prevent osteoporosis and other adverse postoperative issues.

## 1. Introduction

Obesity has become a worldwide epidemic accompanied by significant adverse health effects [1]. Psycho-social factors and genetic dispositions, in addition to an unfavorable lifestyle, constitute causal factors for obesity [2]. Lifestyle changes, medications, and surgery are strategies for the management of obesity. In recent years, the therapeutic use of bariatric surgery has increased substantially [3]. Individuals with very high body mass index (BMI > 40 kg/m^2^) or obese subjects with BMI in the range of 35–40 with one of the obesity-associated complications are elected for surgery. Additionally, patients with type 2 diabetes (T2DM) who have insufficient blood glucose control on traditional therapy may be referred to bariatric surgery even when having a BMI value below 35 kg/m^2^ [4].

The two most common methods for bariatric surgery today are Roux-en-Y gastric bypass (RYGB) and sleeve gastrectomy (SG). In SG, the stomach size is reduced, which leads to early satiety and reduced food intake. The RYGB method involves reducing the stomach size as part of an operative creation of a bypass outside the absorptive gut segments [5]. Both the SG and the RYGB procedures have side effects. Several observational studies have reported an increased risk of fractures postoperatively [6]. This has been related to the observations of mineral and micronutrient deficiencies [2,7,8]. The different types of surgeries impact the absorption of nutrients differently. Being purely restrictive, SG predominantly affects the absorption of vitamin B_12_ and also vitamin D, while RYGB has a more profound impact on the absorption of macro- and micronutrients, including lipid-soluble vitamins [8]. Osteopenia and osteoporosis have especially been attributed to RYGB because of a substantially reduced absorption of calcium and vitamin D [8,9]. However, other deficiencies might also play a role in bone loss. The issue of nutrient deficiencies and supplementation in patients who have undergone bariatric surgery has been addressed by several authors [8,9,10]. In addition, deficiencies in vitamin D and other nutrients common in obese individuals *before* bariatric surgery can further aggravate the often-neglected complication of postoperative osteoporosis.

## 2. Methods

The present narrative review on nutrient deficiencies and osteoporosis is based on a search via Pubmed, Medline, and Google Scholar, in addition to our own research. The keywords used in the search were Bariatric Surgery, Gastric Bypass or Sleeve Gastrectomy, AND Nutrients or Osteoporosis. The search was limited to papers in the English language published in the period 2000–2022. The review summarizes some of the micronutrient deficiencies following bariatric surgery that can lead to bone loss. Postoperative osteoporosis associated with the deficiencies is discussed.

## 3. Pathogenic Aspects of Bone Loss and Nutrient Deficiencies

Osteoporosis is a serious and debilitating disease characterized by reduced bone mineral mass and changed bone microarchitecture. According to WHO [11], a diagnosis of osteoporosis is made when the bone mineral density (BMD) is 2.5 standard deviations or more below the mean peak BMD for healthy adults, as measured by dual energy X-ray absorption (DXA). “Established osteoporosis” is diagnosed in individuals with at least one fragility fracture. A less severe condition is osteopenia, which is defined as a bone mineral density that is lower than normal but not low enough to be classified as osteoporosis [12]. Usually, osteopenia increases in severity with age and is most prevalent in postmenopausal women. Osteopenia is often referred to as “pre-osteoporosis”. More than 200 million people are presumed to be affected by osteoporosis worldwide [11]. Hip fracture is a common clinical manifestation. At least 2 million hip fractures have been estimated to occur annually. Extrapolations indicate that this incidence will increase approximately fourfold during the next 50 years [11]. Apart from hip fractures, other debilitating fractures, such as a compression fracture of a vertebra, represent severe consequences of osteoporosis. These complications underscore the severity of osteoporosis when they occur after bariatric surgery.

In obese individuals who have undergone bariatric surgery, osteoporosis can be caused by nutrient deficiencies, especially deficiencies in calcium and vitamin D, which may lead to hyperparathyroidism with calcium mobilization from the skeleton. Among the aggravating causes of osteoporosis are systemic inflammations, and it is known that pro-inflammatory cytokines, including IL-6 and TNFα, in addition to nutrient deficiencies, can contribute to bone loss in other gut disorders such as celiac disease and inflammatory bowel syndrome [13]. Although obesity is accompanied by low-grade inflammation, post-operative osteoporosis in bariatric patients has been solely attributed to nutrient deficiencies. Various micronutrient deficiencies appear to be involved in postoperative bone loss, as discussed in the paragraphs below.

### 3.1. Nutrient Deficiencies in Morbid Obesity before Bariatric Surgery

Obese individuals often have reduced sunlight exposure due to low outdoor activity. Even without surgery, secondary hyperparathyroidism may develop in obese individuals due to low vitamin D levels combined with a deficient calcium status [14,15,16]. It has also been reported that increased fat deposits in the body may work as a sink and reduce the circulating levels of fat-soluble vitamins such as vitamin D [17]. In addition, an increased intake of sugar-rich or phosphate-containing soft drinks reduces milk consumption and thus leads to a lower intake of milk-derived nutrients, including calcium and vitamin D [18]. In the European population, obese individuals have lower levels of circulating vitamin D than people of normal weight [19], and similar findings have been reported in the USA [18,20]. Vitamin D deficiency in obese individuals may accelerate bone loss [21] and may also lead to a reduced peak bone mass. Circulating levels of magnesium, another nutrient important for bone health, are also reduced in obese individuals [22,23]. Most dietary magnesium comes from vegetables [24], and individuals with obesity and overweight often consume fewer fruits and vegetables [25]. Here, it is important to highlight that the intake of energy-dense food with a high calorie content does not necessarily bring about adequate quantities of vitamins, minerals, and trace elements.

**Figure 1:**
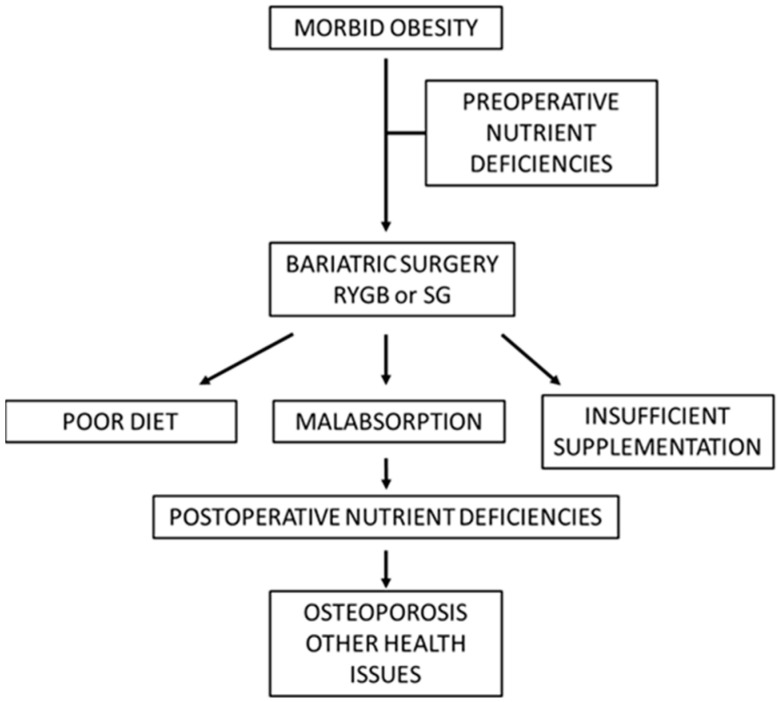
Postoperative development of nutrient deficiencies complicated with bone loss and/or other health issues (schematic). Among other health issues are anemia, fatigue, poor wound healing, hair loss, and neurological symptoms.

Upon referral to bariatric surgery, obese individuals are often diagnosed with preoperative vitamin D deficiency and increased parathyroid hormone levels [26]. Furthermore, a Norwegian study also found subnormal preoperative levels of zinc, copper, and manganese [27]. Similar observations were made by Ernst et al. [28], who measured a broad spectrum of micronutrients in 232 individuals before bariatric surgery and found that about 25% displayed zinc deficiency, and about 25% presented with a subnormal vitamin D status. A study by Schweiger et al. [26], including 114 patients, found that among several deficiencies, about 25% had subnormal values for folic acid. It is common for morbidly obese subjects to present with micronutrient deficiencies [29] (Figure 1), which should be taken into consideration when planning bariatric surgery [30].

### 3.2. Postoperative Vitamin Deficiencies Which Can Lead to Bone Loss

Early studies revealed that patients who had undergone bariatric surgery very often became deficient in several nutrients (Figure 1). While only a mild protein deficiency was observed after SG and RYGB [31], several research groups found that bariatric surgery could aggravate or precipitate vitamin D deficiency and lead to bone loss [32]. At least 20% of operated individuals suffered from vitamin D deficiency after 2 years, and the incidence could be significantly higher after 5 years, despite the recommended supplementation with about 15 μg vitamin D_3_ daily [33,34]. This indicates that higher doses might be needed to prevent vitamin D deficiency. In general, malabsorptive procedures such as RYGB could result in a more severe reduction in vitamin D levels than restrictive surgery [34] due to the bypass of intestinal segments with vitamin D absorption [35]. However, restrictive methods such as SG could also precipitate vitamin D deficiency [36]. Deficiencies in other fat-soluble vitamins, such as vitamins A, E, and K, are also frequently observed after bariatric surgery [37].

According to a recent systematic review by Sherf-Dagan et al. [38], patients who have undergone malabsorptive surgery, including RYGB, are also at a high risk of developing a vitamin K deficiency. In addition to reduced blood clotting, this deficiency is considered one of the causes of increased bone resorption postoperatively due to reduced activation (carboxylation) of osteocalcin. This protein is produced by osteoblasts, but its carboxylation and ability to bind calcium in bone depend on an adequate supply of vitamin K [39,40,41,42]. Of further interest, it seems that vitamin D and vitamin K might have a synergistic effect on bone mineral density (BMD) [39,42]. Vitamin K occurs in the diet in two major forms: Vitamin K1 (phylloquinone) is the major form and is derived from plant-based food, mainly from green leafy vegetables such as spinach and cabbage [43]. Vitamin K2 (menaquinones) contributes less to vitamin K intake in Western diets. Remarkably, it is still not generally established whether postoperative vitamin K supplementation is required.

It is well known that postoperative deficiency of vitamin B_12_ can lead to anemia [44,45]. Still more importantly, vitamin B_12_ deficiency has been associated with osteoporosis, which was reported in a systematic review by Macedo et al. [46]. Today, postoperative vitamin B_12_ supplementation is routinely recommended following bariatric surgery [47]. Folic acid deficiency (vitamin B_9_) is also reported after bariatric surgery [48,49]. Notably, folate deficiency has been associated with reduced bone health due to disturbed collagen crosslinking [50]. This deficiency can easily be corrected by oral supplementation, routinely administered as a multivitamin tablet taken daily. However, non-compliance with recommended vitamin supplements may be a problem, as in our studies in Norway, only about 80% of the subjects followed the recommendations [27,51]. Vitamin C deficiency is also observed in bariatric subjects postoperatively, partly due to poor diet after surgery. It might affect the skin and the connective tissue, in severe cases leading to scurvy [52].

As bariatric surgery is associated with an increased risk of several vitamin deficiencies of importance for bone health, the use of adequate supplements represents an indispensable strategy to combat the associated health complications [53,54]. Some comments on postoperative deficiencies of minerals and trace elements are relevant due to their relation to deteriorated bone health postoperatively. 

### 3.3. Postoperative Mineral and Trace Element Deficiencies Which Can Accelerate Bone Loss

It is the mineral content that gives bones their rigidity. Bone contains a crystalline hydroxylapatite phase embedded in a flexible organic matrix, the latter being composed of collagenous fibers. A simplified expression of the composition of the mineral crystals is given by the formula: Ca_10_(PO_4_)_6_(OH)_2_. However, each of these interacting chemical components may, to some extent, be exchanged with other elements in vivo, which may strengthen or weaken the bone tissue [55]. Thus, calcium ions can be substituted by other bivalent metal ions, such as magnesium, strontium, or zinc ions. Furthermore, the first-line therapeutic agents, viz. the bisphosphonates, that exert protective effects on bone tissue may interact with calcium at the superficial bone layers, exchanging with the phosphate anions. The univalent hydroxyl moiety in hydroxylapatite may, to some extent, be substituted by other anions, such as fluoride. It is apparent that dietary intake, together with intestinal absorption, influences the concentrations of minerals and trace elements detected in human bone. Some reported concentrations of elements in human bones are given in Table 1.

Several metals other than calcium have been reported to be important for bone health (Table 1), either as components in the crystalline phase or as parts of metalloenzymes. It is well known that magnesium, among other functions, is built into the crystal structure of bone. Magnesium deficiency may contribute to the development of osteopenia and osteoporosis [27,57], indicating that calcium supplementation should be combined with an adequate magnesium intake. In addition, copper (Cu), manganese (Mn), and zinc (Zn) are essential for bone formation [58,59]. It may be suggested that supplementation of zinc, and also of copper and manganese, could add to the beneficial effect of calcium supplementation in selected subjects with osteopenia or osteoporosis. Not much is known about the role of iron (Fe) in bone metabolism, although it has been observed in mice that iron overload induces increased bone resorption [60].

Here, some additional comments on postoperative deficiencies of calcium, copper, and zinc are relevant due to their central roles in deteriorated bone health after bariatric surgery. Postoperative deficiencies of these elements are more pronounced after RYGB than after SG [27,61].

#### 3.3.1. Calcium

It has been reported that hypocalcemia after RYGB occurs in 5–25% of patients [61,62,63]. It is important to note that vitamin D deficiency can exacerbate calcium deficiency, since vitamin D is necessary for intestinal calcium absorption [64]. Thus, a strategy based on adequate supplementation of both calcium and vitamin D is crucial to reduce the risk of postoperative bone loss and osteoporosis [64,65,66]. In addition, it appears that *vitamin K* has a profound impact on the kinetics of calcium [67] by acting as an essential component for the synthesis of the metal-chelating groups on Ca^2+^-binding proteins that transfer calcium into the hydroxylapatite crystals [40].

#### 3.3.2. Copper

Copper acts as a cofactor for several enzymes associated with intracellular functions, such as superoxide dismutase, cytochrome C oxidase, and amine oxidases. The copper and zinc-dependent superoxide dismutases are important for bone metabolism [68], and copper deficiency can affect bone health [68,69]. The RYGB bariatric surgery can precipitate copper deficiency since, postoperatively, the food bypasses its absorption sites in the upper part of the small intestine [27]. It has been reported that copper deficiency affects at least 15% of individuals after RYGB surgery [27,69,70].

#### 3.3.3. Zinc

The role of zinc in bone formation is being increasingly appreciated [69]. Loss of zinc in the urine of women with osteoporosis was suggested as an early biomarker for post-menopausal osteoporosis, and supplements of adequate amounts of this trace element to improve bone density have been recommended [71]. A deficiency in zinc after bariatric surgery has been observed by several researchers [27,69]. Zinc is absorbed in the proximal intestine and, bypassing the absorption sites, leads to poor status. Research shows that up to 40–50% of patients experience zinc deficiency after RYGB [27]. Adequate zinc supplementation after bariatric surgery is essential and is of particular importance for bone health [71,72]. It has been suggested that zinc can exert a protective action against bone loss by suppressing osteoclastogenesis via the downregulation of RANKL/RANK, resembling the action of the drug denosumab [72]. Despite prescribed supplementation, several studies have shown that zinc levels can decrease significantly after RYGB surgery [27,73,74,75].

## 4. Postoperative Osteopenia and Osteoporosis

Even in the absence of bariatric surgery, obese individuals may have an increased risk of hip fractures, as recorded after the age of sixty [76], which has been attributed to a deficient vitamin D status [20,21]. Bariatric surgery, which uses stomach size reduction, such as SG, in the same way as the use of proton pump inhibitors, seems to represent an increased risk of reduced bone health due to impaired micronutrient uptake [77]. However, osteoporosis after bariatric surgery has particularly been observed after RYGB and is considered to be related to substantially reduced absorption of both calcium and vitamin D [39]. Bypass of the duodenum and proximal jejunum, which are gut segments with particularly high calcium absorption, is thought to predispose patients to postoperative osteoporosis [78]. Insufficient status of zinc and vitamin K may, in addition to that of vitamin D and calcium, be responsible for accelerated postoperative bone loss and an increased rate of fractures [40,55]. Supplements of essential trace nutrients, including zinc, to improve bone density have been recommended [71]. Copper is also linked to bone metabolism, which has been noticed under severe copper-deficient conditions [69,79]. However, copper deficiency primarily affects collagen metabolism [79]. It has been suggested that supra-nutritional supplements with zinc [72] could add to the beneficial effect of vitamin D and calcium supplementation in selected subjects with osteopenia.

**Figure 2:**
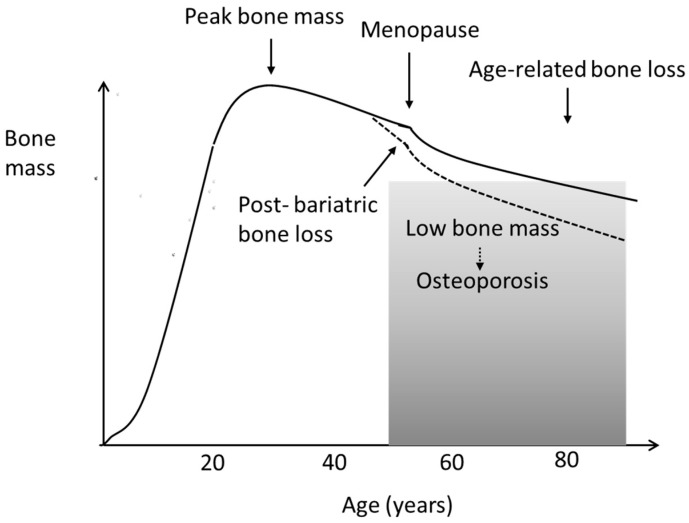
Age-related loss of bone mass in women (schematic). In both women and men, age-related bone loss can be accelerated by bariatric surgery.

As regards the pathogenesis of osteoporosis, it is of interest that a significant loss of bone mineral density (BMD) in the hip and lumbar spine was observed during the first postoperative year by Hofsø et al. [80] in a recent Norwegian study. The bone loss occurred especially after RYGB, despite adequate supplementation with vitamin D_3_, about 40 μg/day, after dose adjustments guided by plasma values [80]. Their observations could indicate that either the weight loss itself or an insufficient status of vitamin K or zinc could play a role in bone deterioration [27,40]. It is known that weight loss after bariatric surgery takes place during the first postoperative year, after which the weight is kept essentially stable during the next couple of years [44]. However, the bone loss also continues during the second postoperative year [81], indicating that the weight loss itself does not cause a decline in bone mineral density (BMD) [40]. Interestingly, Brzozowska et al. [81] found that the forearm BMD loss also continued during the third year after surgery, although the lumbal bone loss plateaued after two years, strengthening the hypothesis that the mechanical unloading of the skeleton does not fully account for the postoperative development of osteoporosis. Neither Hofsø et al. [80] nor Brzozowska et al. [81] assessed the status of vitamin K or zinc in their studies. Several other researchers have reported an increased risk of fractures after bariatric surgery [82,83,84]. Nevertheless, in many cases, postoperative bone loss may pass unnoticed in many cases until it is later interpreted as accelerated age-related bone loss, also called primary osteoporosis (Figure 2).

Altogether, postoperative osteoporosis appears to be the complex result of several nutrient deficiencies. Based on the studies reviewed here, it is strongly recommended to undertake follow-ups with determinations of BMD (densitometry) and biomarkers of bone metabolism, as well as nutrient assessments for all bariatric patients at regular intervals. At the very least, BMD determination should be conducted as part of a routine examination two years after the bariatric surgery. The most widely used biomarkers for bone formation and bone resorption are P1NP (propeptide of type 1 collagen) and CTX (the C-terminal telopeptide), respectively, the blood plasma levels of both these markers being typically increased during ongoing bone loss [85]. For prevention, the bisphosphonate zoledronate, when given as monotherapy, has been found insufficient [86], which further underscores the importance of individualized evaluation and specific dietary supplements. Measurement of 25-OH-vitamin D at the preoperative control is highly recommended, and adequate supplementation should be initiated. At the postoperative follow-ups, it is advised to conduct an extensive examination, including determinations of calcium, albumin, phosphate, and PTH, as well as bone densitometry in individual patients.

In this context, it must be underlined that *pregnancy after bariatric surgery* requires special attention. Considering that most of the treated patients are women of reproductive age and that weight loss has a positive impact on fertility, the effects of bariatric surgery on pregnancy—and on post-pregnancy health—deserve particular attention [87]. Women of reproductive age should be provided counseling for contraception and preconception after surgery. Oral contraceptives are considered less effective in women after bariatric surgery due to poor absorption. Women should delay pregnancy until 12 months *after* bariatric surgery when optimal weight loss has been achieved [88]. As for the monitoring of nutritional status, a dedicated team of doctors and nutritionists should follow up on the status of pregnant women after bariatric surgery. Adequate use of multivitamins and other nutritional supplements is essential [53]. Because unspecific symptoms such as fatigue, nausea, and abdominal pain might be related to postoperative nutrient deficiencies, they should be taken seriously. Deficits in essential nutrients such as folic acid, zinc, calcium, and the vitamins K and D represent a main concern during pregnancy, since these nutrients are essential for the normal development of the fetus, as well as for the maintenance of the general and bone health of the mother [50,53,79]. Upon the follow-ups *after delivery*, it should be taken into account that the mother might experience an increased risk of pregnancy-/lactation-associated osteoporosis, implying that biomarkers of bone health should be routinely checked [89].

## 5. Conclusions

Morbid obesity is a disorder of considerable energy surplus. In recent years, bariatric surgery has gained increasing attention as an effective therapy. However, the most common types of bariatric surgery, RYGB, and SG, are associated with postoperative complications resulting from deficiencies in several essential minerals and micronutrients. Neglect of the serious complication of bone loss and osteoporosis, which appear to occur more frequently after RYGB than after SG, should be avoided by regular postoperative examinations. A lack of essential nutrients may severely compromise bone metabolism and other physiological functions. Individuals undergoing bariatric surgery should be carefully checked before and at regular intervals after the surgery to disclose nutrient deficiencies and bone loss. Increased attention should be paid to the prevention of the postoperative development of osteoporosis. In this respect, the possible benefit of supplementation with sufficiently high doses of vitamin K and zinc should be highlighted in future research, since previous studies have reported postoperative deficiencies. Considering the costs of adequate follow-up to avoid complications after bariatric surgery, further studies are needed to evaluate which categories of obese individuals might benefit most from lifestyle counseling and in which cases surgery with close follow-up is to be recommended.

*Limitations:* Most of the studies on nutrient deficiencies after RYGB and SG have been conducted on populations from the USA and Europe, with a few studies from Asian countries. In all studies, there has been a preponderance of women. Since there are differences in dietary patterns around the world, some caution is necessary regarding interpretations and generalizations. In particular, we still have too few studies on the impact of bariatric surgery on vitamin K status, and the role of vitamin K in postoperative bone health.

In conclusion, bariatric patients need regular postoperative controls, including bone densitometry and assessments of bone metabolism biomarkers, and individual treatment with dietary supplements to manage nutrient deficiencies. Current international guidelines agree that multivitamins and calcium supplementation with added vitamin D are required for all post-bariatric patients. In addition, healthy nutrition with adequate and balanced vitamin K and zinc intake is recommended to prevent deficiencies and for general health.

## Figures and Tables

**Table 1:** Reported concentrations of selected elements in human bones [56] (values in mg/kg unless otherwise indicated).

Element	
Ca	150–250 g/kg
Mg	100–400 mg/kg
Zn	50–260 mg/kg
Cu	0.2–26 mg/kg
Mn	0.1–8 mg/kg

## Data Availability

Not applicable.
